# Complete Atrioventricular Block with Intact Retrograde Conduction in Cardiac Rhythm Management Devices: Implications of the Phenomenon

**DOI:** 10.19102/icrm.2019.100408

**Published:** 2019-04-15

**Authors:** Sumer Dhir, Grant E. Gould

**Affiliations:** ^1^University Of Kansas St. Francis Heart and Vascular Center, Topeka, KS, USA; ^2^Abbott Laboratories, Chicago, IL, USA

**Keywords:** Cardiac pacemaker, complete heart block, implantable cardioverter-defibrillator, pacemaker-mediated tachycardia, retrograde conduction

## Abstract

Intact retrograde ventriculoatrial (VA) conduction in the presence of complete atrioventricular (AV) heart block has been well-documented in the past. We sought to describe the prevalence and clinical significance of intact VA conduction accompanied by complete antegrade AV block in patients with implanted cardiac rhythm management (CRM) devices. During routine follow-up of CRM devices in our device clinic, 42 patients were found to be in a state of complete heart block. All patients presented in sinus rhythm. The patients’ underlying rhythms were tested with the inhibition of pacing and documented AV dissociation. Subsequently, retrograde VA conduction was tested with ventricular pacing. In the 42 patients with complete heart block as the underlying rhythm, five patients demonstrated retrograde VA conduction. In conclusion, the prevalence of intact of VA conduction was 11.9% in our study. The implications of this phenomenon can have noteworthy clinical significance. The occurrence of pacemaker-mediated tachycardia and repetitive nonreentrant VA synchrony are discussed herein. All patients, even those with a device indication of complete heart block, should be tested for retrograde conduction at implantation and during routine follow-up.

## Introduction

In patients with complete atrioventricular (AV) block, the presence of intact retrograde ventriculoatrial (VA) conduction has been well-documented in the past.^1,2^ In a large study of 432 patients with complete AV block, 14% of the study population demonstrated intact VA conduction.^3^ Other studies show a prevalence of about 15%.^4,5^ However, the majority of these investigations were conducted between 30 years and 40 years ago and, to the best of our knowledge, there have been no recent updates published on this subject. Furthermore, the implications now have more clinical importance given the high volume of cardiac device implantations, especially with the use of dual-chamber pacing.^6^

The structure of the AV node has been postulated for the past several decades. Clinical research supports the hypothesis of the existence of bidirectional pathways, one for antegrade conduction and one for retrograde conduction.^7–10^ Thus, the local conduction disease of one pathway can lead to unidirectional block, allowing for conduction to be present in the opposite direction.^11,12^

In this current evaluation of patients with complete antegrade AV block, we assessed the prevalence of intact retrograde VA conduction and the consequences as related to the management of cardiac rhythm management (CRM) devices.

## Methods

Within a device clinic population of more than 1,100 patients, 42 patients were found to be in complete AV block during a routine evaluation of their CRM device between September 2012 and April 2013. The 42 patients identified for possible participation in the study included 27 males (64.3%) and 15 females (35.7%). The mean age of the study population was 77.4 years (range: 29–101 years). All patients presented in sinus rhythm. Patients presenting with atrial arrhythmias were excluded from this study. Sensing, impedance, and pacing thresholds were performed in all patients. An underlying rhythm test was performed using a mode of DDI or VVI at a rate of 30 bpm to 35 bpm or inhibition of pacing, if that was all the functionality that the specific device allowed for. Then, a ventricular lead pacing test was performed, while an atrial electrogram or device marker channel was observed. Retrograde VA conduction was analyzed to verify or exclude VA conduction.

## Results

Of the 42 patients who presented in complete AV block as the underlying rhythm, five patients (11.9%) demonstrated intact retrograde VA conduction.

## Discussion

Advancements in CRM technology as well as the volume of device implantations for arrhythmias and heart failure are growing. A United States study evaluated trends from 1993 through 2009, using the Nationwide Inpatient Sample to identify permanent pacemaker implants. The authors of this study found a 55.6% increase in permanent pacemaker implantation, with a total of 2.9 million patients receiving devices, during this period. The utilization of dual-chamber devices increased from 62% of all implants in 1993 to 82% in 2009.^13^ Similar studies have been performed across the globe and have confirmed this trend.^14^

Hence, with the increase in the use of pacing applications, it is interesting to assess the prevalence and understand the clinical implications of complete heart block with intact VA conduction in the current era.

A significant and one of the most widely known complications is pacemaker-mediated tachycardia (PMT). PMT is a macroreentrant tachycardia that employs the device’s right ventricular lead as the antegrade limb and the patient’s intrinsic VA conduction as the retrograde limb of the tachycardia circuit. The retrograde P-wave is sensed by the device and AV delay timing is reached. In effect, the device now paces the ventricle, with the signal again propagating retrogradely to the atria and being sensed by the device, creating an endless loop.^15^ Normally, in patients with complete AV block, one would not expect this tachycardia because of the conduction disease, but it may occur in such patients. With the ongoing evolution in device algorithms, efforts to prevent PMT and intervene when PMT may occur have improved in the last 20 years. These include the ability to program a post-VA refractory period (PVARP), adaptive PVARP, atrial pacing after a premature ventricular contraction (PVC), and mode-switching for a single beat after a PVC.^16^ Withholding a ventricular pacing impulse and delivering an atrial impulse in its place as an alternative to terminate PMT is also an option. However, perhaps the former is more advantageous to the patient to avoid PMT, with proper programming of the device.

A closely related arrhythmia instigated by dual-chamber devices is a repetitive nonreentrant ventriculoatrial synchrony (RNRVAS). RNRVAS is often initiated with patient activity and an increase in the atrial pacing rate or loss of AV synchrony. It equally results in a functional undersensed retrograde P-wave, due to falling into the PVARP. In this instance, the device delivers a functional noncaptured atrial pacing impulse because of the relatively long atrial effective refractory period. Then, the paced AV delay time is reached, prompting the device to deliver a ventricular pacing impulse and then propagating the impulse back to the atria via retrograde conduction.^17^ A case study suggests that programming a rate-response PVARP with the shortest minimum duration may quickly terminate and prevent sensor-driven RNRVAS.^18^ Further setting modifications should perhaps be considered, such as shortening the paced AV delay and/or decreasing the lower pacing rate to accomplish a similar desired outcome.^19^ This study also suggests an increase in the frequency of RNRVAS because of the prolonged AV timing delays in response to reducing right ventricular pacing.^19^ Sequentially, this timing increase is more relevant because of the increase in dual-chamber implantations, as noted above.

Both of these cardiac device-mediated tachyarrhythmias that disrupt AV synchrony can affect intrinsic timing and lead to pacemaker syndrome. Pacemaker syndrome is defined as an phenomenon of nonphysiologic timing between atrial and ventricle depolarization, resulting in patients displaying a wide range of symptoms.^4^ These include dizziness, weakness, presyncope, or syncope and a greater than 20 mmHg reduction in systolic blood pressure when the patient receives VVI pacing versus atrial pacing or sinus rhythm.^20^ These adverse symptoms were significant when VVI-mode devices were utilized in patients in normal sinus rhythm with complete heart block and exhibiting intact VA conduction. Now, with maintaining AV synchrony with dual-chamber pacing as a common practice and with proper programming of the device ensured, the risk of pacemaker syndrome is minimized.^20^ However, it is worth mentioning about this in the present discussion because of its relation to both device-mediated tachycardias.

### Study limitations

In most patients, the VA conduction times were only tested at 600 ms (100 bpm), and it is possible that intact VA conduction exists at a slower rate. It may have been beneficial to use decremental pacing at a slower rate to distinguish absolute VA dissociation and true VA block.

One specific device model of one manufacturer does not have the functionality to test the underlying rhythm by temporary pacing at a nontracking mode and at a lower rate. In the particular device, the underlying rhythm is tested instead by turning the pacing off and, in the present study, ventricular pacing was suspended for around three seconds without any ventricular response to atrial activity. However, it is possible that pacing suppression of the ventricle occurred, resulting in slowing of the intrinsic response for greater than the three seconds that pacing was inhibited.

Furthermore, concealed extranodal accessory pathways cannot be ruled out as a path for VA conduction.^1^

It was not noted in the data collection process whether any of the participants who demonstrated intact VA conduction also experienced episodes of an endless loop tachycardia or RNRVAS or not. This was an observational study that assessed the prevalence of intact VA conduction in patients demonstrating complete AV block and the implications of the use of CRM devices in a certain subselection of patients. It may be interesting to review the device interrogations of the affected study population to see if there was evidence of these pacemaker-instigated tachyarrhythmias.

Additionally, this was a single-center study with a relatively small patient sample size.

## Conclusion

The prevalence of retrograde VA conduction in patients with complete antegrade AV block in our study was 11.9%. All patients, even those with a device indication of complete heart block, should be tested for retrograde conduction at implantation and during routine follow-up. The implications of intact VA conduction in patients in complete heart block with CRM devices can have noteworthy clinical significance. Further clinical investigations with larger patient populations may be needed to address this issue adequately.
